# Policy impacts in dynamic relation to food, income, learning and security: COVID-19 lockdowns in a Nigerian Agrarian Community

**DOI:** 10.1007/s10668-024-04938-2

**Published:** 2024-05-09

**Authors:** Grace Oluwakemi Awosanmi, Ayodeji Fisayo Afolayan, Mia Perry, George Olusola Ajibade, Sunday Adesola Ajayi

**Affiliations:** 1 African Institute for Science Policy and Innovation, Obafemi Awolowo University, Ile-Ife, Nigeria; 2 Sustainable Futures Global Network, Nigeria Hub, Obafemi Awolowo University, Ile-Ife, Nigeria; 3 Institute for Sustainable Development, First Technical University, Ibadan, Nigeria; 4 School of Education, University of Glasgow, Glasgow, Scotland; 5 Department of Linguistics, Obafemi Awolowo University, Ile-Ife, Nigeria

**Keywords:** Covid 19 pandemic, Participatory research, Lockdown policy, Livelihoods, Education

## Abstract

*Whose Crisis? The Global COVID-19 pandemic from the perspective of communities in Africa* is an international research project that aims to investigate and represent the diverse experiences of the COVID-19 pandemic from those marginalized by mainstream media and policy influence. This article focuses on the multidimensional effects of the generalized lockdown policy in an agrarian community in Nigeria. The project engaged participatory and culturally responsive adaptations of qualitative methods including participatory engagement and individual and group discussions with purposively selected community members. This relational research practice is supported by a Systems Thinking approach to data analysis. In particular, a Causal Loop Diagram (CLD) is used to analyse and visually present the relationships between various elements (variables) of the research context (the system). This study reveals the interrelated effects of the COVID-19 prompted generalised lockdown policies on livelihoods, education, health, and security in rural Nigeria. Although the lockdown policy was intended to curtail the impact of COVID-19, it had severe unintended consequences, exposing weaknesses in the social support system and threatening the foundations of the agrarian community of this study. This article culminates in recommendations for participatory and culturally responsive approaches to future policy formulation.

## Introduction

Nigeria’s population is almost equally split between urban and rural dwellers (Macrotrend, 2021). Presently, the agrarian sector, dominated by subsistence, peasant, or small-scale farmers, provides over 80% of the total food produced in the country (Mgbenka et al., 2016; Omodero, 2021). This sector is also the main provider of raw materials to major markets for domestic manufacturing (Vincent et al., 2021; Yakubu & Aderonmu, 2010). Itagunmodi, in the Southwest of Nigeria, is a community typical of many rural agrarian settings in the region and is the focal location of this study. This community is engaged as part of a wider research project that seeks to amplify the voices and experiences of under-represented and under-served communities in Africa to contribute to the understanding of Global health in a pandemic context. The larger project, *Whose Crisis? The Global COVID-19 pandemic from the perspective of communities in Africa,* has exposed otherwise unseen aspects of the lives of people affected by the global shifts and generalized policies prompted by COVID-19 (Perry et. al., 2021).

Rapid research has already been published on the effects of lockdowns on food security (e.g., Eze, et al., 2021), mental health (e.g., Huang et al., 2020), education (e.g., UNESCO, 2020) businesses and income (e.g., WorldBank & NBS, 2020) and global economy (e.g., Elliot, 2020), however a full understanding of the plural perspectives and experiences of this time requires interdisciplinary and culturally responsive research. Research that genuinely reflects the complex lived realities of those made most vulnerable in Nigeria in this time also requires trusting partnerships between community leaders, a range of community members, and visiting researchers. A commitment to this relational approach underpins the study and directly informs the concluding reflections and recommendations relating to genuine community participation in policy development and implementation.

After a brief review of the COVID-19 pandemic—with a particular focus on the materialisation of governance measures in Nigeria in the first two years of its emergence—this article moves on to explore the research methodology in more depth, outlining the physical and demographic contexts of the study as well as the place-based approach to research practice. Finally, these contexts of the place and the pandemic are put into dynamic relation to the related areas of livelihoods, health, education, and security.

### COVID-19 and lockdowns

The coronavirus, SARS-CoV-2 (COVID-19), was detected in 2019 and caused a severe pandemic of a highly contagious respiratory illness. It has led to the death of over 6 million people around the globe, as well as health complications in others who have survived the illness (Casecella et al., 2022). Nigeria recorded her first case of COVID-19 on February 27, 2020 (Nigerian Centre for Disease Control, 2020), one month later, a global pandemic was declared by the World Health Organisation (WHO). A range of restrictions were put in place by governments around the world to contain the contagion and reduce congestion in healthcare systems (Fakir & Bharati, 2021). These restrictions included a series of non-pharmaceutical interventions identified as lockdowns (Regmi and Lwin (2021). By April 2020, almost 3.9 billion persons in more than 90 countries were commanded to stay at home or be “locked down” by their governments (Sandford, 2020).

As the spread of the virus progressed and increased rapidly within each state in the country, WHO stated that Nigeria and 13 other African countries were at particularly high risk (Ezigbo & Ifijeh, 2020). In response to this high-risk status, the Federal and State governments followed the example of other nations and declared directives for lockdowns to curtail the rates of infection. On March 20, 2020, the Osun State government prohibited public gatherings of more than 50 persons in schools, churches, and mosques (Abubakar, 2020). By March 24, 2020, it barred weekly markets indefinitely (Adedeji, 2020). There was a follow-up announcement by March 31, 2020, instructing a total lockdown restricting intra and inter-state movements, with the exemption for those in essential services (NBC News, 2020).

Although frontline health care is a global priority, attempts to meet the health challenges of a pandemic exist within a fragile ecosystem, particularly in agrarian communities in Nigeria. COVID-19 highlighted existing inequalities and made very clear the bio-social nature of the disease (Gibbon et al. 2020) wherein biological vulnerabilities are created through socio-economic inequalities. A lack of contextual, faith, traditional, cultural understanding, involvement of local communities, and recognition of national priorities can negatively affect the health interventions and outcomes that arise from COVID-19 and thus distort health policy and governance (e.g., the widespread imposition of “lockdown” policies). Millions of Nigerians observed the lockdown announced by both the federal and state governments, which placed multiple hardships on those who lacked basic provisions such as income and food for their families during these circumstances (Campbell & McCaslin, 2020; Ewang, 2020). The restrictions impacted all sectors across the socio-economic strata of the population.

## Methodology

This case study took place as part of a larger multi-site case study project that spanned Nigeria, Botswana, Uganda, Malawi, and Eswatini. Common objectives and research questions led to the individual design of methods to respond to the needs and expectations of communities in conjunction with the expertise and capacities of the researchers. The research questions that guided the overarching project include: (1) What are the lived experiences of, perspectives on, and responses to, COVID-19 in vulnerable communities in sub-Saharan Africa?; (2) How can perspectives be shared in participatory and collaborative ways to share Northern and Southern expertise, resources, and engagement?; and (3) What can be achieved when the voices of under-represented and under-served communities in Africa are amplified, in terms of Global Health in a pandemic context?

Decolonial methodologies broadly describe research practices that resist the imposition of colonial epistemologies, that deny the tendency of extractive research practices, and that honour the values, knowledge systems, and practices of the participants or place of the intended research (see for e.g., Patel, 2016; Tuhiwai Smith, 2012). This work takes many forms, dependent on the particulars of place, topic, and people involved. Participatory, cultural, and transdisciplinary methods are typical of decolonial research, and unsurprisingly the research is often led or guided by researchers indigenous to, or in close relationship to the place. In this research presented here, the core research team in the Nigerian context are Nigerian researchers that span a range of disciplinary expertise (an ethnologist, a rural sociologist; a policy analyst; an agriculturist; and systems thinking analyst^1^), and are local to the participating community. Our community-based approach called for a careful establishment of relationships with community leaders and a negotiation of engagements with community groups (accounting for cultural preferences as well as public health guidance relating to human interactions at the time). Working in collaboration, community members of Itagunmodi participated in the research in various ways, prioritizing their needs and perspectives. We used methods that spanned informal conversation, story-telling, and participatory observations, as well as more formal methods that could be recognised as interviews and focus group discussions.

For this Nigerian case study, a Systems Thinking (ST) analytic approach was taken up to provide a framework with which to engage with a complex array of ideas and support a coherent visual portrayal of interconnectedness and interdependency (Pattenden, 2021). This approach prevents the simplification of issues that can occur with research in a singular discipline or from a singular perspective. ST is a way of making sense of the complexity of intersecting factors, considering the whole situation and relationships within it, rather than parts. In this case study, ST enables a depiction of the effects of the COVID-19 prompted policies on an agrarian community as a “system”.

Across a range of engagements, researchers with participants foregrounded the complex lived experiences of communities and households in Itagunmodi, whilst ensuring a wide global witnessing of these voices. Lived experience, as well as an increasing body of research, confirms the interrelations of local and global; community and national; social and ecological systems and practice—on the individual, environment, and public health (Amusan & Agunyai, 2021; Elliot, 2020; Eze et al., 2021; Lehtonen et al., 2018). However, as Pattenden (2021) also noted, common issues impact individuals or individual systems in distinct and unequal ways. The proceeding section will describe the specific contexts of this study (and “system”).

### Study area

**Fig. 1:**
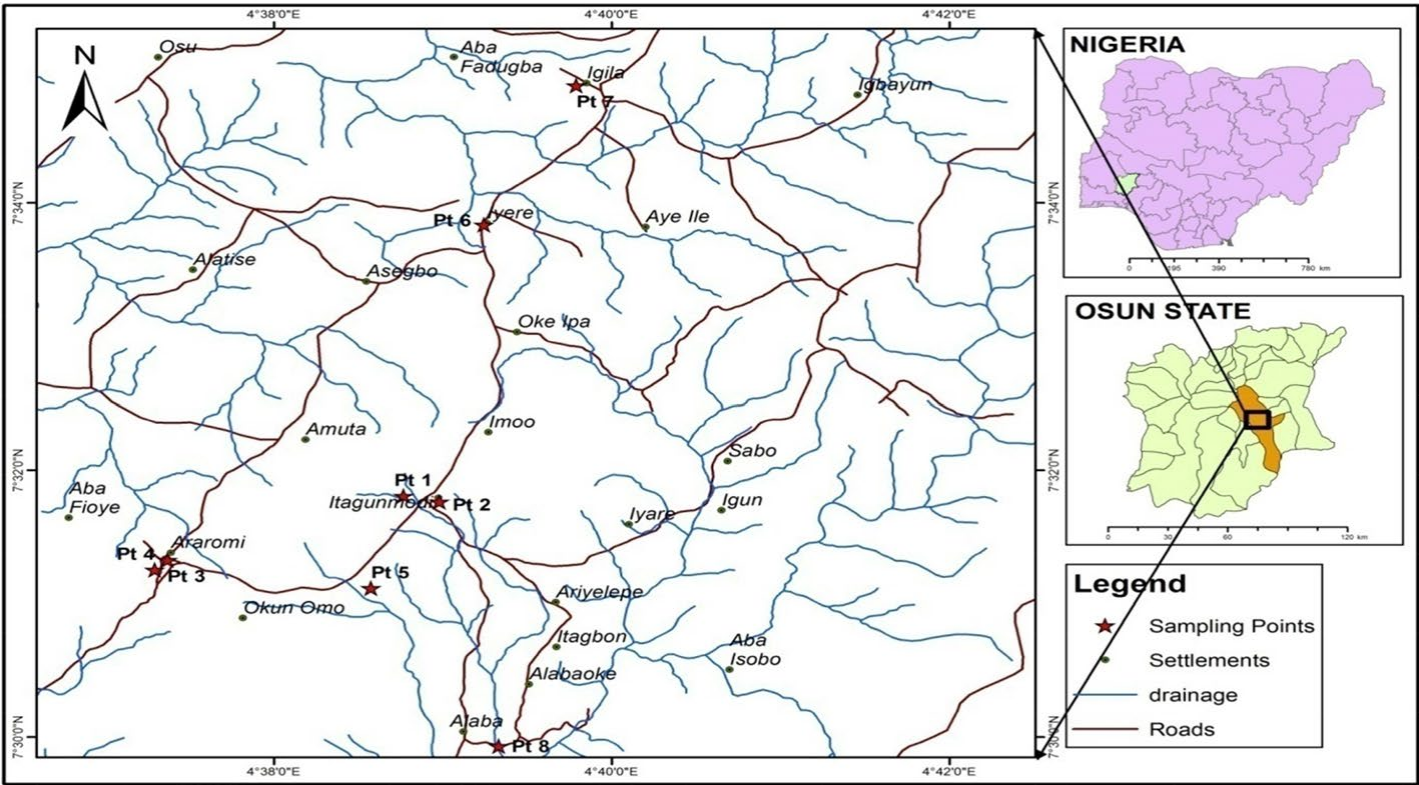
Map of Itagunmodi

Itagunmodi is an agrarian community located in the Atakunmosa West Local Government Area of Osun State, in the South-west geopolitical zone in Nigeria. It lies on latitudes 7° 13′ 26″ and 7° 48′ 28″ N and longitudes 3° 52′ 0″ an 4° 47′ 59″ E, and 347 m above sea level (see Fig. 1). The community is situated in a rainforest ecological zone with abundant rainfall from March to October and dry spells from November to February (World Weather Online, 2021). This environment provides opportunities for cropping patterns for such crops as cocoa, plantain, oil palm, maize, cassava, citrus, and vegetables. The temperature ranges between 27 and 32 °C. The inhabitants of the area are estimated at 12655, comprising indigenes and non-indigenes (SFA, 2021). The majority of the inhabitants are artisans, food vendors, traders, subsistence farmers, and labourers, who earn and depend on their daily wages to feed themselves and their families. Most of these people do not have savings and their daily earnings serve as the only means of sustenance. Rural communities in Nigeria, such as Itagunmodi, are characterized by poor infrastructure including untarred and dusty roads; poor health centers; a high rate of illiteracy; and high rates of poverty (Adelabu, 2019). As would be expected, the key occupation of the residents at Itagunmodi is farming of both staple and cash crops. The community has four streams namely: Omi Agunmodi, Omi Eleyinju, Omi Eku, and Omi Aayo. The only source of potable water is a functional borehole and the Omi Eku River. This is a result of the deposits of mineral sediments by the artisanal miners into the streams and rivers making the water slightly acidic and contaminated (SFA, 2021). Thus, this has led to a scarcity of potable water in the community. The community has only one primary health care centre, a public primary, and a secondary school.

Food security in this region of Nigeria is far from stable. The recent upsurge in global hunger data by the Food and Agriculture Organization (FAO) has been attributed to the restriction of trade and food-producing activities due to the effects of the pandemic (Mardones, 2020). Individuals who are food secure do not live in hunger or fear, thus food insecurity is described as the inability to access the right amount of nourishment (FAO, 2006). The incidences of austere food scarcity among the Nigerian populace have been on the increase, and the number of people that experience hunger increased from 6.4% in 2014 to 9.1% in 2019 (Varrella, 2020), and about 49.7% of this population are rural dwellers. The incidence of food uncertainty in low-income urban households and rural areas in Nigeria is at 79% and 71% (Akerele et al., 2013). The COVID-19 lockdown aggravated food shortage in Africa (World Food Programme, 2020). Similarly, increased prices of food led to lower quality and quantity of food intake at the household level which is hazardous to households’ food security (Ojo, 2020).

### Data collection

The study was carried out between September 2020 and June 2021. With appropriate approval secured from the village head and council members, a town hall meeting involving both indigenes (the traditional dwellers and owners of the land) and non-indigenes (dwellers that have migrated to the community) was organized to create broad awareness about the study. At the meeting, community members were openly invited to participate, the only criteria being their own willingness and availability. Data were collected using interrelated methods including participant observation, open and focus group discussions, story-telling, and in-depth one-to-one interviews.

*Focus Group Discussion* Group discussions were organised into 3 sessions. This allowed for 1 female group and 2 male groups of 7, 7, and 9 participants respectively. Collectively, the 23 participants included a mix of artisans, farmers, and entrepreneurs. The participants were within the age group of 20–60 years, 21 of them were married and 12 were single; 5 had tertiary education, 4 secondary education, 10 primary, and 4 no educational qualification. Among these participants, 18 are from the Yoruba ethnic group, 2 from Igbo, and 1 from Hausa.

*One-to-one or key informant interviews* 5 females and 4 males took part in in-depth interviews with members of the research team. The individuals were from various backgrounds and had different roles in the community. These included leaders from the community, youth, union associations, and religious. The researchers are very familiar with the general culture and language of the community; thus, they were able to use open-ended questions, which allowed the participants to think, reflect, and express themselves freely. The voice notes were audiotaped, transcribed, and translated into English for accessible engagement with research analysis across the diverse research team. Although lots of information was garnered from the community members, the authors were primarily concerned with the evidence of a broad range of impacts of the COVID-19 lockdown on the community.

### Data analysis

**Fig. 2:**
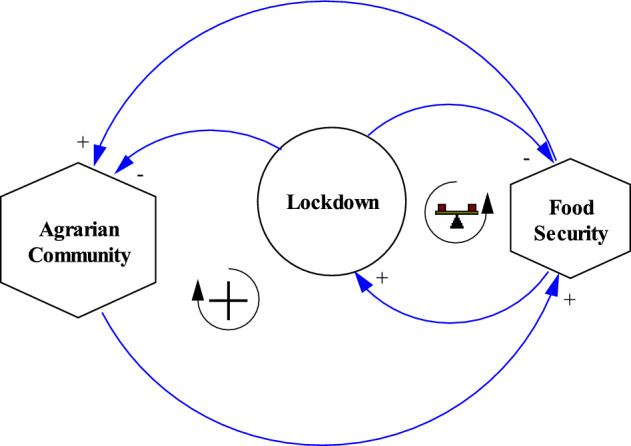
Interconnections between the Agrarian community, lockdown, and food security

Cognisant of the complex socio-economic situation explored in this study, a System Thinking (ST) approach was deployed to evaluate the complexity of the experiences shared by the community members. The study framework was based on ST principles and practices. These principles revolve around the notion of “holistic” understanding, i.e., the notion that the essentials of a system can best be understood by also observing adjacent components and their connections. This “whole process” was thus viewed as a “system”, representing the interconnections between the “variables” considered for the study, as shown in Fig. 2.

**Fig. 3:**
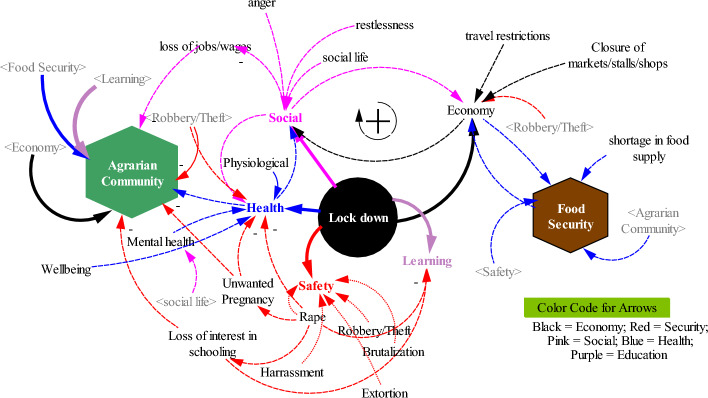
Implication of lockdown policy on an agrarian community and food security in the country

A system is a network of multiple variables linked to one another through causal relationships that express some sort of behaviour, which can only be explained through observation of the whole (Toole, 2005). The variables of primary interest in this analysis were termed “agrarian community”, “lockdown^2^” and “food security” (Fig. 2). Each variable was examined equally. So, the system (e.g., agrarian community) is more than a collection of its parts (e.g., lockdown and food security) (Arnold & Wade, 2015). Furthermore, System Thinking sheds light on the interconnections that occur in the system through the use of causal loop diagramming, as shown in Fig. 3.

**Fig. 4:**
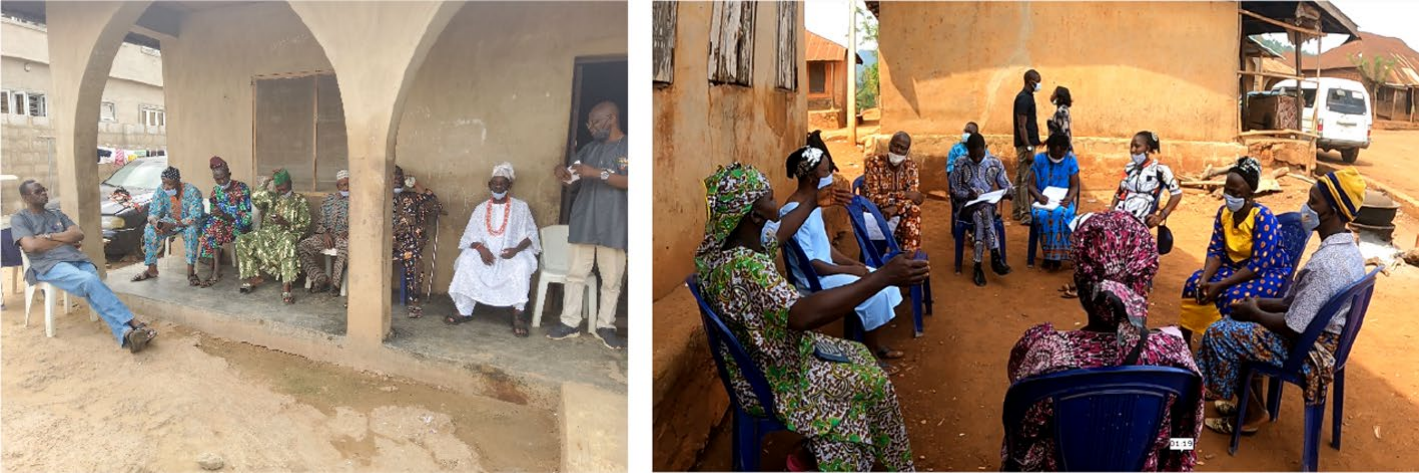
Sample of male and female group discussions

**Fig. 5:**
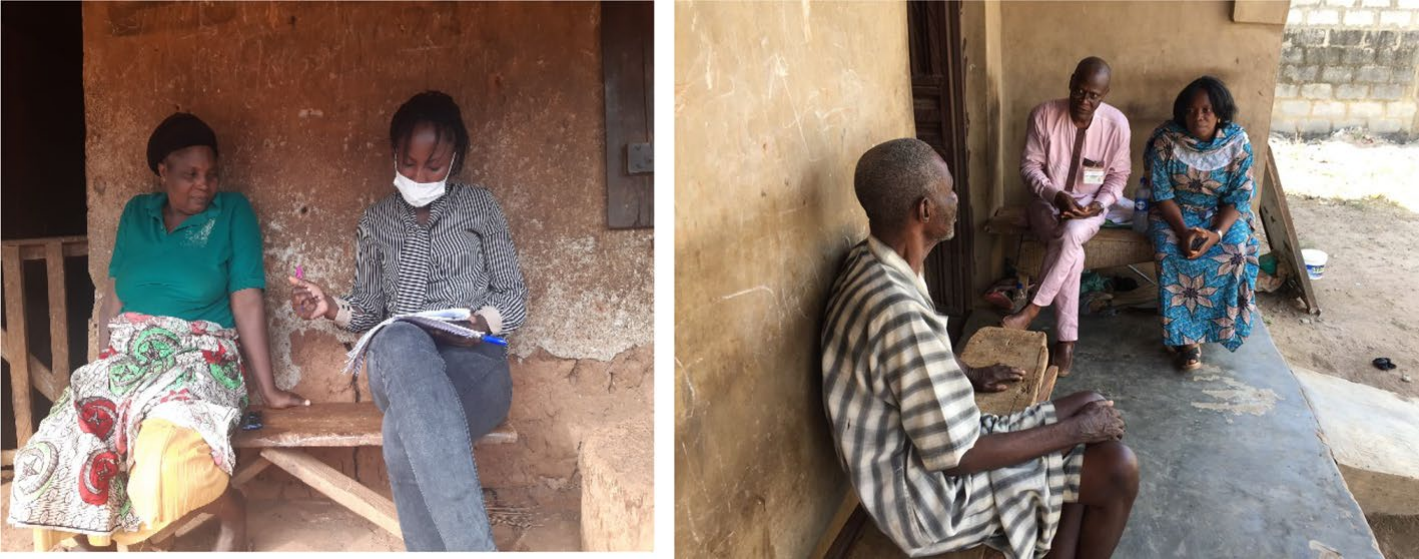
Sample of in-depth interview

The CLD represented as Fig. 3 portrays qualitative data (generated with participatory observation and discussions) in relation to the key variables identified in the system. The accuracy and precision of the qualitative data is based in the iterative and participatory approach of the research practice. For example, interactions with community members in group discussions were revisited and clarified in individual interviews (see Figs. 4 & 5). Issues raised in individual encounters were related and compared to observations recorded in other community engagements. The CLD in Fig. 3 captures the complexity and dynamics of the social systems of Itagunmodi community and uncovers various underlying patterns and trends. Of course, it does not present a complete picture, so the CLD model is used to expand the researchers’ mental models and boundaries of thinking beyond the parochial (Perlow et al., 2002). Pragmatically, we use the CLD to give an overview of the effects of the lockdown on the agrarian community. Below, detailed discussion and data excerpts add specific content to this overview.

## Results and discussion

This section elaborates on the causal loop diagram, presenting further findings from interactions with inhabitants of Itagunmodi. The discussion focuses primarily on the ways in which the generalised lockdown policy related to and impacted livelihoods, health, education, and security.

### Livelihoods

The lockdown had a wide range of direct effects on communities, including, on social lives, economy, health, safety, and learning. This in turn affected the agricultural practices in the community and consequently, food security in the country. In terms of social life, as is indicated in Fig. 3, anger, quarrels, restlessness, and loss of wages and jobs increased as gleaned from the respondents. Also, in terms of economy, there were closures of shops, markets, and stalls, travel restrictions, and rampant cases of robbery and theft reports. The examples, regarding the social and economic life of the inhabitants in the community, are illustrated by some responses to these situations:

“I have never been in a police cell before, but I was because of the lockdown. I was bored at home and decided to go out, unfortunately, I and many others were caught by the law enforcement agents. I had to stay in the cell until I was granted bail”.

“I was always angry with my children who often complained about the small quantity of food that they were served; I became more irritational during the restriction and often picked up arguments with my husband”.

“We could not go out then. There are some people that if they don't go out to work in a day, it will be difficult to survive and there were some people that died of hunger during this period”.

“We did not have anywhere to go, no jobs, we were struggling to survive. Many people died as a result of hunger because we were told to stay indoors. People that died were said to have COVID, but that was not true. People died as a result of hunger”.

“The problem I encountered during that period was no work; there was no movement due to lockdown. There was no one for us to transport, we were rendered jobless. I was borrowing money from friends, taking food on credits like rice, gari, etc. that helped”

“It affected those people in the educational sector gravely, majorly private schools. Because most of these people do not have another source of income except these teaching jobs”

The examples above reflect experiences of both informal workers and professionals. With little to no institutional support from employers at the time, these professionals such as teachers, health care workers, and business owners were left without income, thereby unable to contribute to the informal work sector. The widescale restriction of movement also made it difficult for people such as “Okada”^3^ riders, labourers, taxi drivers, artisans, hawkers, and food vendors to go about their daily sustenance activities. The restricted movement led to the loss of daily and monthly incomes, and job losses further impoverished the people. Moreover, the inability of people to engage in intra and inter-work and business transactions exacerbated the challenges of food shortages and access to other essential supplies. This resonates with findings from COVID-19 studies in India, Kenya, and Uganda that claim that the pandemic resulted in increased unemployment, decreased income for daily labour, increased food insecurity, depletion of savings, and reduction of relief materials (Kesar et al., 2020; Harris et al., 2020; Janssens et al., 2021; Kansiime et al. 2021). Nigeria’s National-level COVID-19 Impact Monitoring Survey confirms that 79% of respondents, including those in agrarian and non-agrarian communities, noted a reduction in their household income since the occurrence of the pandemic (WorldBank & NBS, 2020).

### Health and well-being

The lockdown policy affected the physiological and emotional health of the people leading to the health implications indicated in Fig. 3. Community members explained that they had to change their dietary patterns to mitigate the reduction or loss of income. As illustrated in their responses below, a low-quality carbohydrate-based diet became the main source of nourishment to survive for many, despite the impacts of this on their health and that of their children.

“Eating well is like a 5-star achievement during this period, As a father and a commercial driver. I could not afford èlùubọ́ (Yam Flour) as a proper meal for my family. Instead, I opted for half-nutritious dried láafúun (grounded cassava flakes) gathered from my little farm just to eat. Providing for my kids was hard because as the day-to-day earner that I am, if I didn't work per day, we would all be starved. Sadly, my kids developed Kwashiorkor from this unhealthy diet”

“When the lockdown started, I had to go to my farm with my wife to harvest some cassava which was processed to láafúun (grounded cassava flakes). It was this láafúun that we were making to amala to eat morning, day and night. Luckily for us, it was during the rainy season, we went to the farm every 3 days and when we saw some vegetables, we harvested them and used them to eat amala. It was this same meal we were rotating until we ran out of èlùbọ́ then I had no option but to start using the money that I had secured for business”

“I eat only once a day and due to this, I had difficulties in sleeping which prompted me to visit a doctor who checked my BP and told me it was high and that I have to stop worrying if I value my life”

People compromised in different ways to survive during this time, including eating only once or twice a day or eating low-nutrient foods. Responses revealed that the restrictions also took a toll on mental health.

“...I pity the women who went to borrow from to feed their family from cooperatives most. These organizations were merciless and not lenient with the payback procedures and time. The women out of their frustration and desperation engage in sexual immorality (prostitution) to pay back because the organizations insisted that all borrowed loans must be repaid regardless of the circumstance and within the set time. Such loans can only be cancelled or overlooked when the borrower is dead and the organisation also confirms by seeing the corpse”

“Some people were even forced to go and collect loans from microfinance banks due to lack of money but since they could not go out to work, they did not have rest of mind because they could not pay back”.

The restrictions led to many people buying food on credit, borrowing from friends, or using loans taken out for business purposes from microcredit organizations, to purchase daily food. The participants indicated that those of them who borrowed to survive invariably suffered anxiety related to debt. The findings align with Koos et al. (2020), who found that most rural households adopted coping strategies such as a reduction in certain types of consumption, the sale of productive assets, and borrowing at high-interest rates to mitigate income shocks. These tendencies put people in danger of malnutrition and related health challenges (WHO, 2019). The use of sex in exchange for food or money was widely reported during the lockdown period, and became a survival mechanism for the women who had families and relied on their daily earnings. Amusan and Agunyai (2021) also found that many vulnerable households across African states engaged in transactional sex and debt to buy food during the lockdown.

### Learning and teaching

The impact on formal learning practices in Nigeria has been primarily affected by school closures, as indicated in Fig. 3. School closures contributed variously to a loss of interest in schooling, unwanted pregnancies, theft and robbery. These conditions all contribute to a negative impact on formal and informal education across the community. The normal, formal educational system in Nigeria, from primary school to tertiary level, is carried out through face-to-face practice. Traditionally throughout this learning period, learners are not introduced to the use of any electronic teaching or digital equipment (Eze et al., 2021). Eze et al. (2021) noted that primary and secondary school learners in Nigeria are traditionally not allowed to bring or use any digital devices such as a phone or computers in the schools. Thus, without easy recourse to online learning, the educational system was almost entirely halted during the lockdown.

“the school-aged children in the community were kept at home with their parents without any educational engagement for 6 months since all schools in the country were on lockdown”.

“schools were shut down; my children were home because they had nowhere to go, no school, no home lesson”.

“the lockdown also made students lose interest in their studies”

There were exceptions to the shutdown of schools with schools that introduced the use of laptops and mobile phones for the delivery of learning. However, only a few parents could afford to provide supporting infrastructure for learning at home (Tadesse & Mu-luye, 2020). A widescale lack of sufficient digital devices along with poor and unstable internet connections resulted in a low uptake of any online education provision provided by schools or the State government. Furthermore, education programming on the radio and television was also only partially accessible due to an unreliable electricity supply during this period and a lack of money to purchase batteries for radios.

Consequently, the closure of schools and all other face-to-face educational learning activities placed additional burdens on the impoverished families who were already economically strained as a result of the restrictions from the lockdown. To cope with the broader situation, school-aged children were deployed to “hawk” for their parents.

...I sneak to the farm to harvest some of my farm produce in the mornings before the security forces come into the community, and when they disperse in the mid-afternoon I ask my children to quickly hawk some of these produce in exchange for other food items or money.

### Agricultural production and supply chain

The lockdown had a direct negative impact on food security as shown in Fig. 3. The lockdown period coincided with the cropping season and harvesting by farmers in the community. Usually, this season involves preparation work such as ploughing and crop planting, which the restrictions hindered. Also, cash crops for harvest during this period could not be transported to markets for sale due to the restrictions of movement and closures of markets.

“The COVID-19 period spoilt a lot of things which translates into money for us e.g. selling of cocoa beans, at the period when we're expected to start selling it out. We were told that the country that will buy it cannot do so because of the restrictions”.

“The restriction of movement affected us, those middlemen we do supply our produce to, to help us resell, told us that it is after two days of selling our farm produce they will give us money all because security agents are not making the marketing process easy, because they get stopped on the road. Some of our plantains got spoilt due to their perishable nature which leads to a reduction of profit for us”.

“I had a bitter experience during the restriction. It occurred at a time when we ought to prepare ourselves and our customers ahead of the planting season. Owing to the restrictions placed by the government which was enforced by placing security agents everywhere, we could not go anywhere. Those who want to travel to get agro-chemicals to preserve their farm produce could not travel. It brought a great fall on our economy”.

Thus, restricted movements affected the cultivation, management, harvesting, and transportation of farm produce from the community. The purchase of agricultural inputs like seeds, fertilizers, herbicides, and pesticides was inaccessible; the cultivation of annual crops like maize, cassava, and yams was hindered; and crops, such as bananas, cocoa, vegetables, and fruits could not be harvested. Notably, the transportation of farm produce to designated sales points was forbidden, and anyone caught was severely punished and the goods destroyed. Generally, the shortage of agricultural inputs and access to markets were critical constraints to agricultural activities in the community. These issues were particularly critical due to the absence of food storage facilities in the agrarian community. Our findings are in line with Pu and Zhong (2020) and Rasul (2021) who assert that agricultural supply chains were disrupted, and the challenges of food security and sustainable livelihoods in developing countries were compounded. Conclusively, the constraints to agricultural production and its supply chain, and the loss of revenue to farmers have direct impacts on poverty rates and future food security. To cope with the situation, the farmers resorted to exchanging their harvested products for other food types.

### Safety and security

The lockdown policies contributed to a lack of physical safety in communities during this period. As shown in Fig. 3, the participants revealed cases of harassment, extortion, and brutality during the restrictions. Generally, rural communities are perceived to have low crime rates due to the family and religious values embedded within them, coupled with socio-cultural conventions and practices. However, as the lockdown progressed, issues of social unrest escalated and harassment, brutality, rape, robbery, and theft began to occur more frequently in the community.

“I remembered asking my son to take me on Okada to get food supplies from a neighbouring community, we had to start our journey early to avoid and escape the law enforcement agent on our way. Unfortunately, I had to part with money in about seven different points before we were permitted to pass”.

“Homes and shops were broken into, to cater away foodstuff and valuable items, and we had reported cases of rapes in the community”.

### Policy impacts in dynamic relation

The above analysis demonstrates the adverse effects of lockdown policies on the lives and livelihoods of Itagunmodi community members. The loss of livelihood, disruption in agricultural production and supply chains, lack of safety and security, disruption in learning, and health implications were the primary and interconnected issues that emerged. The governance decisions were quick responses to global reports on the spread of the virus, mortality rates, and means of slowing infection rates (Karlinsky & Kobak, 2021). The lockdown enforcement was immediate, with offices, markets, and shops being closed, people staying home, and security agents dispatched to the streets to ensure compliance. However, our research contributes to a growing accounts of community suffering for the sake of policy compliance.

These reactions during the lockdown are articulated in the interconnections within the participatory Causal Loop Diagram (pCLD) (Fig. 3), showing the different obvious (solid lines) and hidden (dotted lines) effects of the lockdown. The agrarian community responded to the lockdown in manners not anticipated. It can be surmised that liquidity was one of the most difficult challenges for members of the community as they were previously daily paid for their goods or services. Nigeria’s financial system was not responsive to this vulnerability in society as credit was not available at this critical time. What could be perceived as coping strategies adopted by many in the community were mostly harmful and predatory such as rape, robbery, quarrelling, fighting, and prostitution.

Further analysis is required to understand the positive lessons and adaptive practices introduced by community members. This includes the adoption of new and innovative food regimes, the renewal of traditional forms of medicine and healthcare to compensate for lack of access to vaccines and pharmaceuticals promoted via global communication channels.

Systems Theory lends a focus to behaviours in a system rather than specific events. Behaviour is a process and the use of the CLD based on ST principles makes plain important feedback loops (Perlow et al., 2002). This foregrounds the *performance* of the system studied. In application, the CLD presents a look at the effects of the lockdown policies on food, livelihoods, safety, and learning. Three major areas of interconnection are summarised with the terms “agrarian community,” “lockdown” and “food security”, each affected by multiple factors. These three areas, interconnected as a system, have a feedback or response loop. The system being studied is represented by the CLD with all its constituent components and interactions. Through the captured interactions, and consequently the feedback loops, the basic structure of the system is revealed. The feedback or response loop shows the process involved in the interconnections within the system. For example, the social life during the lockdown was reflected by some factors like anger, and restlessness, for example. The lockdown occasioned responses (feedback) from the agrarian community members in relation to their multiple areas of impact on their lives. These responses now represent a measure of the *performance* of the lockdown policy in the community.

Therefore, in terms of performance, it can be said that the system, principally the lockdown within it, did not deliver as expected, because it led to unintended consequences (Tenner, 1996), particularly for the agrarian community, in terms of economy, safety, learning, health, and social life. Through the feedback responses, the immediate consequences of the lockdown policy on the agrarian community included, amongst others, hunger, anger, restlessness, theft, and brutality; while the long-term impacts include a loss of interest in schooling, unemployment, and unwanted pregnancies. Immediate and long-term impacts are both explicit and implied.

## Conclusion

The lockdown policies in response to the Global COVID-19 pandemic affected the lives, livelihoods, and health of the world. However, its impacts varied greatly across contexts. In the agrarian community of this study, like many other similar contexts, crop production, small and medium-scale enterprises, and daily jobs are the main livelihood activities. The results of our study show that the lockdown affected livelihoods and caused a detrimental impact on health, well-being, food security, education, and community safety. Also based on the experiences captured from the community, it was revealed that the country's social system in terms of finance, health, and education is weak in response to the needs of the vulnerable in society.

Our research, in alignment with an increasing prevalence of corroborating studies, contributes to a better understanding of the lived experiences and community-level impacts of wide-scale policy implementation during the Global COVID-19 pandemic. It is critical to learn from this work and apply the lessons learned to new practices, planning, and policy formation. To reduce the harm of future global and regional health and environmental threats, participation in policy formation should take place, relating local circumstances and needs with national and global priorities and knowledge. The participation of community actors and civil society organizations in governmental policy formation ensures not only appropriate responses and effective buy-in of the new policy but also a clearer understanding of the scope of a policy domain. For example, this research indicates that a public health policy response, such as a lockdown policy, must be considered about agricultural policies relating, for example, to farm produce storage and market linkages. Similarly, educational policies must be considered relating to, for example, the use of electronic equipment for learning and teaching.

Participatory policy formation can be further enhanced by policy scenario analyses (Balaman, 2019). Scenario analysis in this case could have evaluated the earning power of representative individuals and families at the community level regarding their expenses on daily consumption. This would have indicated required savings or support for precautionary purposes, as well as the length of time these could sustain them. It would highlight mitigation needs such as storage facilities for crop produce to prevent deterioration and digital communication methods for teaching and learning. Based on insights revealed through scenario analysis, the duration of an effective lockdown and the necessary strategies for intervention could be determined a priori.

Combined, participatory policy formation and scenario analysis would likely have cushioned the negative impacts and unintended consequences of the lockdown policy on the agrarian community. Consequently, the study suggests an inclusive approach in future policy formulation An inclusive approach to policy formulation can either be top-down participatory (led by government or delegated organizations) or bottom-up through advocacy by particular stakeholder groups (Rietberge, 2017). In the case of an event such as the COVID-19 pandemic, which is global in nature with local effects, a coordination of both policy formulation approaches is called for. The pandemic serves to make explicit the diverse and substantial local impacts of global events, and therefore the need for a global strategy with local relevance. This is called for not only in relation to global public health, but also in all other interconnected issues including environmental and social equity and economics. In practice this requires wide and genuine stakeholder participation in defining the objectives, modifications and implementations of mutually beneficial policy and related practice.

## Data Availability

The data generation from this project is stored in the Enlighten repository and can be accessed via the following link: https://researchdata.gla.ac.uk/1196/. The data is available via a “Request for Access” function within this repository due to the size of the data files included in relation to the downloading capacities of common web browsers. Further information, selected data, and updated outputs are available via the project website: https://www.whosecrisis.org/. The excluded data includes that which includes highly sensitive material, or that which a participant has requested to exclude.

## Footnotes

1 The service of this analyst was only taken up for the purpose of analysis. The expert did not partake in the study directly.

2 This lockdown was a response to the Covid-19 pandemic.

3 Okada is a local parlance for motor bikes used by individuals for local transportation.
